# A Bibliometric Analysis of the 100 Top-Cited Articles on Vertical Root Fractures

**DOI:** 10.7759/cureus.75300

**Published:** 2024-12-07

**Authors:** Pillai Arun Gopinathan, Ikram UI Haq, Nawaf Alfahad, Saleh Alwatban, Abdullah Alghamdi, Amal Alamri, Kiran Iyer

**Affiliations:** 1 Department of Maxillofacial Surgery and Diagnostic Sciences, College of Dentistry, King Saud Bin Abdulaziz University for Health Sciences, King Abdullah International Medical Research Center, Ministry of National Guard Health Affairs, Riyadh, SAU; 2 College of Dentistry, King Saud Bin Abdulaziz University for Health Sciences, Riyadh, SAU; 3 College of Dentistry, King Saud Bin Abdulaziz University for Health Sciences, King Abdullah International Medical Research Center, Ministry of National Guard Health Affairs, Riyadh, SAU; 4 Department of Public Health, College of Dentistry, King Saud Bin Abdulaziz University for Health Sciences, King Abdullah International Medical Research Center, Ministry of National Guard Health Affairs, Riyadh, SAU

**Keywords:** bibliometric, citation analysis, endodontics, research productivity, vertical root fractures

## Abstract

The aim of this study was to perform a meticulous analysis and bibliometric evaluation of the top 100 most cited articles in vertical root fractures (VRFs). The bibliometric research method included 100 top-cited articles on VRFs retrieved from the Web of Science database. The key terms “vertical root fracture” OR “vertical root fractures” were used to retrieve the required dataset. The salient bibliometric indicators were analyzed. Microsoft Excel version 16 (Microsoft Corporation, Redmond, WA), VOSviewer (v.1.6.10, Centre for Science and Technology Studies, Leiden University, The Netherlands), and the SPSS Statistics version 20 software (IBM Corp., Armonk, NY) were used for data analysis. The top 100 cited articles on VRFs were published between 1977 and 2022, and these articles were cited with an average of 69 citations. A slight rise (27%) in top-cited articles on VRFs was shown in the first half (1977-1999), but a substantial increase (73%) was recorded in the second half (2000-2022). The Journal of Endodontics published around half of the articles (n = 47). The United States contributed the most cited articles, followed by Brazil and Israel, whereas The Netherlands produced the most influential articles. The top-ranked author (Avaid Tames) and the university (Tel Aviv University) belonged to Israel. The most often occurring keywords were also analyzed to identify potential research subjects. There has been a notable increase in the number of highly cited publications (n = 52) about VRFs within the past 13 years (2010-2022). The United States stands out among the top countries due to its dominant overall research output. In the fields of endodontics, oral surgery, and restorative dentistry, this information would be helpful to researchers, practitioners, and academics.

## Introduction and background

Vertical root fractures (VRFs) are a major problem in endodontics and restorative dentistry that frequently results in tooth loss and calls for intricate treatment procedures. Both vital and nonvital teeth may develop a longitudinal split in the root structure, which is a characteristic of VRFs. They are frequently linked to high occlusal stress, prior endodontic treatment, and structural flaws in the tooth from earlier restorations [1-3]. VRFs can have modest clinical presentations, which frequently make diagnosis difficult. Treatment choices may become more difficult due to symptoms such as localized pain, edema, and tooth movement [4]. Cone-beam computed tomography (CBCT), one of the advanced imaging techniques, has made it easier to detect VRFs and better analyze the degree of the fracture and related periapical disease [5]. The therapy of VRFs is still debatable; depending on the kind and degree of the fracture, therapeutic options range from extraction to nonsurgical intervention [6]. According to research, maintaining tooth structure and improving prognosis depend on early detection and adequate treatment [7,8]. Even though VRFs are clinically significant, more research is still required to fully understand their genesis, preventive measures, and long-term effects. This emphasizes how crucial it is for the dental community to continue researching and working together in order to improve the comprehension and treatment of this major clinical issue related to VRFs [9,10].

The need for a thorough grasp of VRFs' occurrence, etiology, diagnostic techniques, and management approaches has grown in the past years as more dental professionals are working on this [7-9]. As far as research is concerned, a useful technique for evaluating the patterns, significance, and latest development called bibliometric analysis, a quantitative approach to examining scholarly literature, is used [11,12]. Bibliometric analysis is a credible and unbiased way to provide quantitative data on a scientific field based on previously published articles, and this method is being applied more in dentistry [13,14]. An article is recognized as one of the most cited when it is cited frequently by other researchers in their academic work [15]. The dataset on the most-cited articles in a given academic arena is provided by the bibliometric analysis, which is based on citation metrics and assists in understanding research trends [16].

Huang et al. carried out the bibliometric analysis of 171 articles on tooth intentional replacement, published from 1964 to 2023 [17]. Authors from 28 countries contributed, but the majority of research came from the United States, followed by China and Japan. Most of the articles were published in the Journal of Endodontics and Dental Traumatology. The study identified the future research themes, and “managing VRFs” was one of them [17]. Another study examined PubMed-indexed articles on “traumatic dental injuries.” Brazil produced most of the research, followed by the United States, Turkey, and India. Israel stood in eighth rank with 2.93% of the total research. Among the most common dental injuries, avulsion was found on top (21%), followed by crown fracture (9.71%) and root fracture (5.90%). More than one-third (33.69%) of the articles were published in Dental Traumatology, and 75% of the articles were related to clinical research studies [18].

The goal of this analysis is to map the 100 top-cited articles on VRFs, highlighting important publications, influential writers, and major themes. One can gain a better understanding of the evolution of VRF research and potential directions for future studies by looking at the geographic distribution of research, the expansion of literature over time, and the networks of collaboration among researchers. In addition to pointing out knowledge gaps, the present bibliometric analysis sheds light on the multidisciplinary character of VRF research, which spans disciplines like materials science, radiography, and dentistry.

## Review

Research methodology

**Figure 1:**
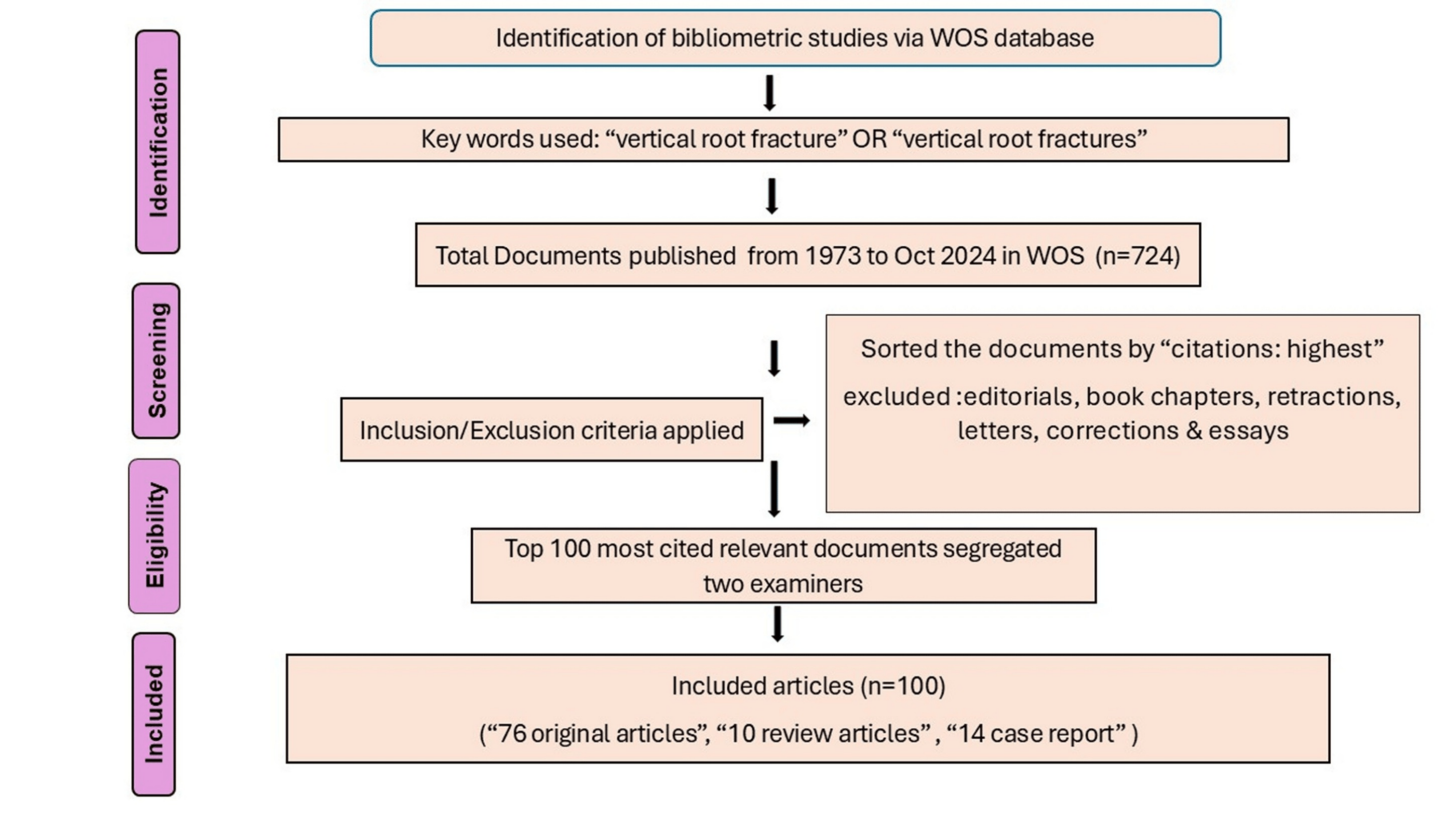
Methodology flowchart WOS: Web of Science Image credit: This is an original image created by the author Pillai Arun Gopinathan

The bibliometric research method was used for the 100 most cited articles retrieved from the WOS database on October 23, 2024. The key terms “vertical root fracture” OR “vertical root fractures” were inserted, and then the documents were sorted by highest citations. The 100 most cited articles were accessed. The bibliometric indicators such as periodic distribution of articles, study design based on level of evidence (LoE), nature of research (clinical and nonclinical), frequently used publication sources, top countries, top institutions, top authors, and authors used keywords were analyzed. The citation metrics and relevance to the targeted subject were the inclusion criteria. The bibliographic details of the top-cited, most appropriate articles on VRFs were downloaded for analysis. The use of publicly available data eliminated the necessity for an institutional review board or ethical sanction for the bibliometric study. Figure 1 shows the flowchart of the methodology followed.

Data analysis tools

Microsoft Excel version 16 (Microsoft Corporation, Redmond, WA), VOSviewer (v.1.6.10, Centre for Science and Technology Studies, Leiden University, The Netherlands), and SPSS Statistics version 20 (IBM Corp., Armonk, NY), i.e., Pearson's chi-square test, were used for statistical analysis. Statistical significance was determined by a p value of less than 0.05.

Results

**Figure 2:**
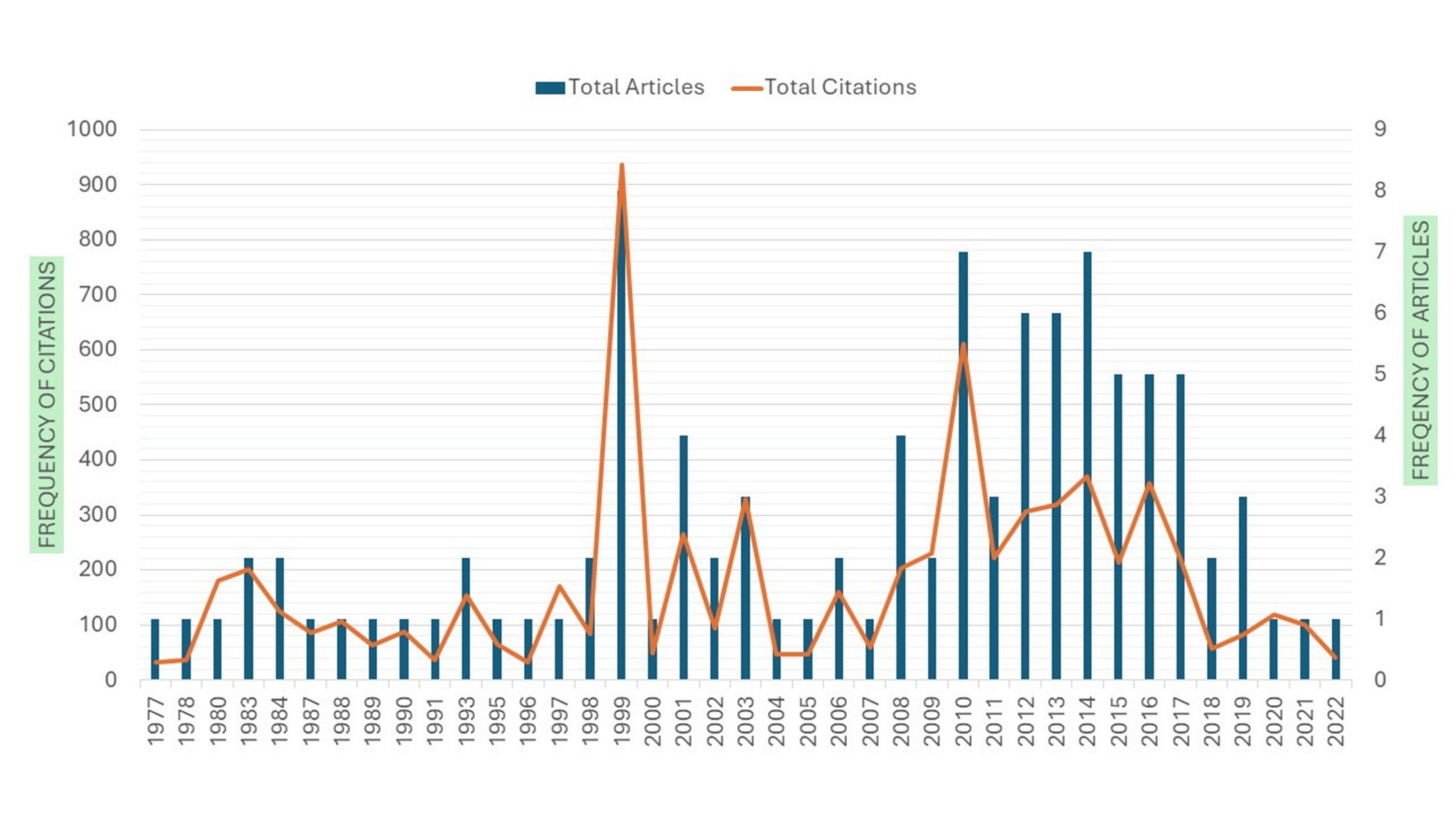
Break down of articles and citation by yearwise distribution Image credit: This is an original image created by the author Ikram UI Haq

The dataset of 100 top-cited articles on VRF spans over 46 years, from 1977 to 2022, providing insights into publication trends and citation metrics across different years. The fluctuation has been observed in the number of articles published, total citations received, and the citation impact over the years, as shown in Figure 2. The year 1999 had the highest total of citations (n = 937), with eight articles published, demonstrating a noteworthy growth in top-cited articles. Citation impact is usually high in earlier years, with numerous occasions exceeding 100 citations per article. There is a clear deterioration in citation impact in the later years, particularly in the 2010s.

There is a significant increase in the top-cited articles from 27 during the first 23 years from 1977 to 1999 to 73 articles in the later 23-year period (2000-2022). This indicates a higher top-cited published after 1999. The increase in top-cited articles after 1999 indicates a growing body of research, which may reflect an expanding field or increased research activity. The articles published in the first 23 years (1977-1999) gained higher citation impact, with an average of 88.96 citations per article, compared with the articles published in the last 23 years (2000-2022), which received an average of 61.66 citations per article.

Table 1 depicts that out of the 100 most cited articles on VRFs, there were significantly more nonclinical articles (n = 72) than clinical articles (n = 28). Clinical studies had an average of 65.04 citations per article, while nonclinical research had a marginally greater influence, as evidenced by the higher citation impact of nonclinical studies, with an average of 70.58 citations per publication. A proportion test was conducted for comparison between clinical and nonclinical studies, as nonclinical studies were cited 5% more than clinical studies and were statistically significant (p value of <0.001) (Table 1). There was an apparent pattern in the distribution of articles by LoE, with lower LoE (III and IV) having more articles and LoE-IV having the most articles (n = 61). LoE-II had the highest citation impact, with 82.67 citations per article, indicating that even though it contains fewer articles, they are quite influential. LoE-III comes in second with 74.95 citations per article, demonstrating its high level of significance. In addition, there was a significant (p value< 0.001) citation impact across different levels of evidence (Table 1).

**Table 1:** Distribution of articles by clinical/nonclinical studies and LoE p value of less than 0.05 is considered statistically significant LoE: level of evidence

Variables	Types of studies	Total articles	Total citations	Citation impact	p value
Clinical or nonclinical	Clinical	28	1,821	65.04	<0.001
	Nonclinical	72	5,082	70.58	
LoE	LoE-I	3	200	66.67	<0.001
	LoE-II	6	496	82.67	
	LoE-III	22	1,649	74.95	
	LoE-IV	61	4,055	66.48	
	LoE-V	8	503	62.88	

Out of the 100 top-cited articles on VRF published in 18 journals, 90% of articles have been published in the top eight journals shown in Table 2. The Journal of Endodontics leads expressively in relation to total articles and citations. This journal has robust productivity and the highest citation count, indicating its reputation in the field of VRF. Oral Surgery Oral Medicine Oral Pathology Oral Radiology and Endodontology had 12 articles and 806 citations, clamming good citation metrics relative to its publication volume, followed by the International Endodontic Journal with the same number of articles (n = 12) but comparatively low citation impact. The Journal of Prosthetic Dentistry, having fewer articles, displays distinguished citation impact, with an average of 108.50 citations per article, demonstrating that its published articles are highly significant. There were 10 journals with one article each (Australian Dental Journal, Endodontics and Dental Traumatology, European Journal of Oral Sciences, Imaging Science in Dentistry, Journal of Dentistry, Journal of Periodontology, Oral Radiology, Oral Surgery Oral Medicine Oral Pathology Oral Radiology, Quintessence International, and Radiology).

**Table 2:** Frequently used journals

Rank	Name of the journal	Total articles	Total citations	Citation impact
1.	Journal of Endodontics	47	3,800	80.85
2.	Oral Surgery Oral Medicine Oral Pathology Oral Radiology and Endodontology	12	806	67.17
3.	International Endodontic Journal	12	692	57.67
4.	Dental Traumatology	7	292	41.71
5.	Dentomaxillofacial Radiology	6	271	45.17
6.	Journal of Prosthetic Dentistry	2	217	108.50
7.	Journal of the American Dental Association	2	128	64.00
8.	Clinical Oral Investigations	2	82	41.00

The authors from 24 countries contributed to the 100 most cited articles on VRF. Table 3 ranks the top 10 most productive countries based on the total number of articles published and their respective citation metrics. The United States leads in total articles (n = 28), while The Netherlands had the highest citation impact, with an average of 108.86 citations per article. Countries like Australia and Israel also illustrated robust impacts, with an average of 89.80 and 85.58 citations per article, respectively. Significant research engagement was demonstrated by countries like Brazil, England, and Turkey. The authors from Germany, Greece, Italy, and Thailand contributed three articles each, while the authors affiliated with Canada, China, Iran, and Sweden produced two articles each. Authors from six countries (Austria, Jordan, Portugal, Russia, South Korea, and Spain) produced one article each.

**Table 3:** Top 10 most productive countries

Rank	Name of the country	Total articles	Total citations	Citation impact
1.	United States	28	1,982	70.79
2.	Brazil	15	690	46.00
3.	Israel	12	1,027	85.58
4.	The Netherlands	7	762	108.86
5.	England	7	437	62.43
6.	Turkey	6	327	54.50
7.	Australia	5	449	89.80
8.	India	4	338	84.50
9.	Japan	4	263	65.75
10.	Taiwan	4	229	57.25

The authors affiliated with 92 institutions contributed to the 100 top-cited articles on VRF, and three-fourths (n = 69; 75%) of the institutions contributed to a single article. Table 4 presents the details of the top 12 most productive institutions, with more than two articles in their contribution. Tel Aviv University leads with a maximum number of articles and high citation counts, resulting in a respectable citation impact (83.64). The articles produced by the authors of the Academic Center for Dentistry Amsterdam gained the highest citation impact (108.85), indicating extraordinary impact relative to the number of articles published. Despite having fewer articles, institutions such as the University of Melbourne, the University of Milan, and the University of Washington exhibit substantial citation impacts.

**Table 4:** Top productive institutions

Rank	Name of the institution	Total articles	Total citations	Citation impact
1.	Tel Aviv University, Israel	12	1,027	85.58
2.	University of Iowa, United States	8	547	68.38
3.	Academic Center for Dentistry Amsterdam, The Netherlands	7	762	108.85
4.	Universidade Estadual de Campinas, Brazil	7	311	44.43
5.	University of Melbourne, Australia	4	378	94.50
6.	King’s College London, England	4	272	68.00
7.	National Taiwan University, Taiwan	4	229	57.25
8.	University of Milan, Italy	3	301	100.33
9.	University of Washington, United States	3	288	96.00
10.	Dicle University, Turkey	3	205	68.33
11.	Federal University of Rio Grande do Sul, Brazil	3	189	63.00
12.	Federal University of Bahia, Brazil	3	160	53.33

A total of 316 authors have been identified, and most of the authors (n = 259; 82%) contributed to a single article. Only 57 authors contributed more than one article, and 20 authors contributed more than two articles each, as shown in Table 5. Avaid Tamse of Tel Aviv University was in first place with 12 publications, and research has been cited with an average of 85.58 citations per article. Zvi Fuss and J. Lustig of Tel Aviv University reached second rank with six articles and had a high citation impact of 104.83. Out of the top 20 authors, seven belonged to Tel Aviv University. This suggests that this institution produces high-quality research in the VRF. Although Ozok and Metska, both from the Academic Center for Dentistry Amsterdam, contributed three articles each, they had the highest citation impact of 133.00. The research community had a wide international presence, as evidenced by other noteworthy universities, including Universidade Estadual de Campinas, King's College London, and the University of Melbourne.

**Table 5:** Top 20 authors having more than two articles

Serial no.	Author	Affiliation	Total articles	Total citations	Citation impact
1.	Tamse, Avaid	Tel Aviv University, Israel	12	1,027	85.58
2.	Fuss, Zvi	Tel Aviv University, Israel	6	629	104.83
3.	Lustig, Joseph	Tel Aviv University, Israel	6	629	104.83
4.	Wesselink, Paul Rudolf	Academic Center for Dentistry Amsterdam, The Netherlands	5	524	104.80
5.	Walton, Richard E.	University of Newcastle, England	5	299	59.80
6.	Freitas, Deborah Queiroz	Universidade Estadual de Campinas, Brazil	5	244	48.80
7.	Lertchirakarn, Veera	University of Melbourne, Australia	4	378	94.50
8.	Messer, Harold H.	University of Melbourne, Australia	4	378	94.50
9.	Patel, Shanon	King’s College London, England	4	272	68.00
10.	Ozok, Ahmet Rifat	Academic Center for Dentistry Amsterdam, The Netherlands	3	399	133.00
11.	Metska, Maria Elissavet	Academic Center for Dentistry Amsterdam, The Netherlands	3	399	133.00
12.	Palamara, Joseph	University of Melbourne, Australia	3	327	109.00
13.	Shemesh, Hagay	Academic Center for Dentistry Amsterdam, The Netherlands	3	317	105.67
14.	Pitts, David L.	University of Washington, United States	3	288	96.00
15.	Mannocci, Francesco	King’s College London, England	3	231	77.00
16.	Tsesis, Igor	Tel Aviv University, Israel	3	229	76.33
17.	Ozer, Senem Yigit	Dicle University, Turkey	3	205	68.33
18.	Jeng, Jiiang Huei	National Taiwan University, Taiwan	3	174	58.00
19.	Kaffe, Israel	Tel Aviv University, Israel	3	170	56.67
20.	Melo, Saulo L. Sausa	Universidade Estadual de Campinas, Brazil	3	108	36.00

Keywords associated with the most referenced papers on VRF are listed in Table 6. While Total Link Strength measures a phrase's overall connection or relevance, possibly revealing how effectively the term ties to other concepts in the literature, Occurrence displays the frequency with which each keyword occurs in the dataset. With 37 occurrences and a total link strength of 117, VRF was the most common term, indicating that it was a major theme in the study. The significance of CBCT in identifying or researching root fractures was demonstrated by its high rank (26 occurrences). The terms “root fracture” and “vertical root fractures” (each with eight occurrences) indicate that different terms were interchanged, which may indicate a particular emphasis.

**Table 6:** Top 10 frequently occurred keywords

Serial no.	Keyword	Occurrence	Total link strength
1.	Vertical root fracture	37	117
2.	Cone-beam computed tomography	26	78
3.	Root fracture	8	30
4.	Vertical root fractures	8	24
5.	Digital radiography	5	16
6.	Endodontically treated teeth	4	12
7.	Endodontics	4	13
8.	Root canal filling	4	16
9.	Tooth fractures	4	11
10.	Fracture susceptibility	2	10

**Figure 3:**
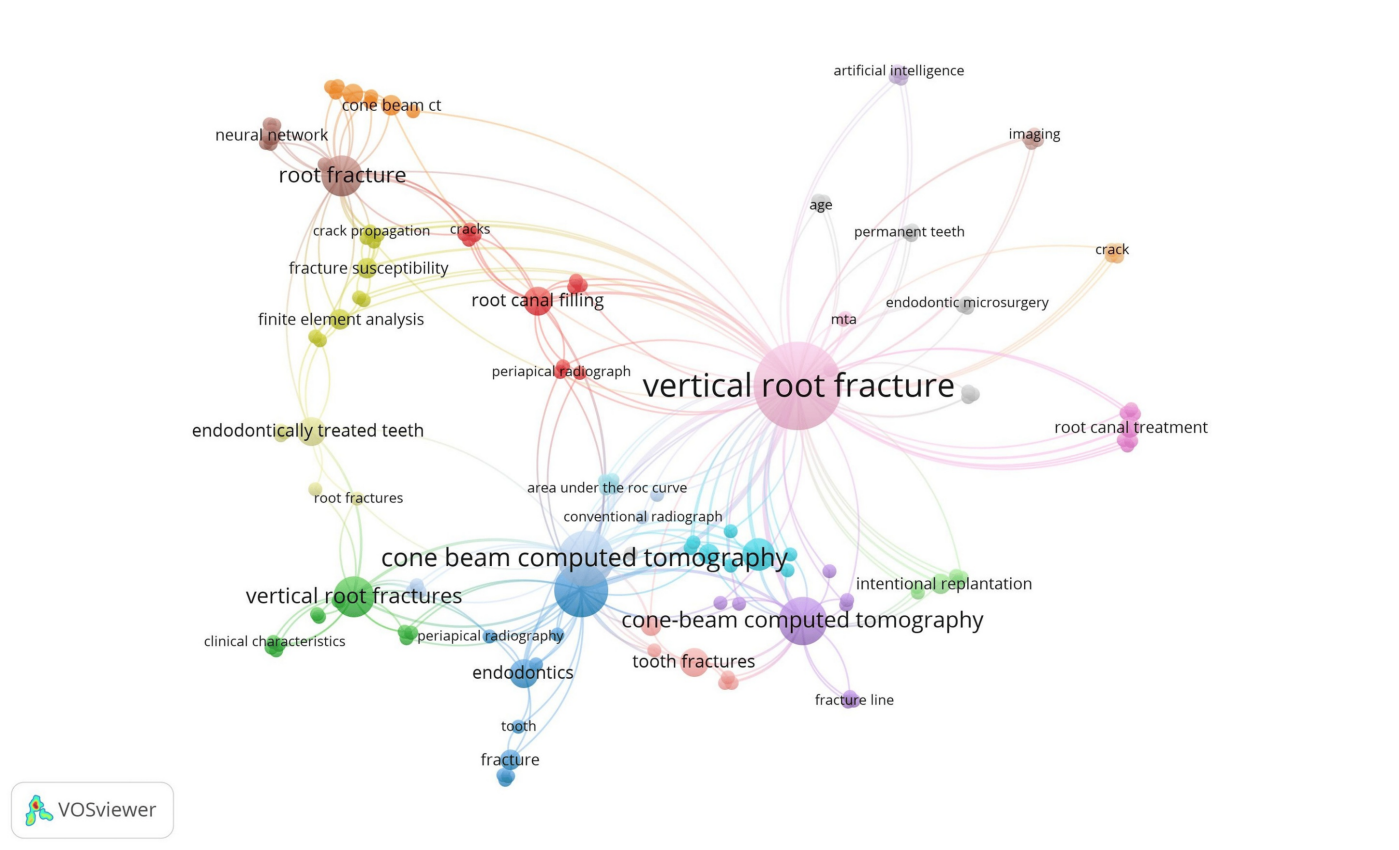
Co-occurrence network of keywords MTA: mineral trioxide aggregate Image credit: This is an original image created by the author Ikram UI Haq

Figure 3, generated with the VOSviewer software, illustrates that out of 146 keywords, 138 keywords are connected in a co-occurrence network, divided into 23 clusters. The top four clusters have ten keywords each; the first cluster consisted of CBCT, cracks, dentin, dentinal damage, mechanical cycling, periapical radiograph, preparation, retreatment, root canal fillings, and root canal instrumentation. The second cluster comprised of these keywords (clinical characteristics, endodontic treatment, flat panel volume detector computer tomography, maxillary premolar, post and core, radiograph, radiography, root canal anatomy, three-dimensional observation, and VRFs), followed by the third cluster (beam hardening artifacts, dental endodontic, diagnosis, digital, endodontics, facture, periapical radiography, tomography, tooth, and vertical fractures) and the fourth cluster (biomechanics, crack propagation, cracked teeth, finite, endodontic posts, finite-element analysis, fracture susceptibility, nickel-titanium file, root-filled teeth, stress distribution, and terminal fracture). The Appendix shows the full details of the 100 most cited articles in VRF, including citation details.

Discussion

Citation metrics are implemented to evaluate published research quality and the ratio of citations, which determines the quality and excellence of the scientific study [11,19]. Citation metrics have been widely employed as a rational indicator of the scholarly impact of work [20]. The present bibliometric analysis offers a variety of findings that are important for exposing the research trends of the top 100 papers on VRFs. The dataset was focused on WOS exclusively; regardless of WOS's restricted coverage compared to that of the Scopus and Google Scholar databases, the overall quality of documents indexed has been higher [21]. One notable strength of this study is that it did not employ filters that restricted language or years except citation counts. This made it possible to thoroughly and broadly analyze every document released up until the search date, which included this theme with the most citations.

In our study, the top-cited articles on VRFs were published in the span of 46 years between 1977 and 2022, and these articles received an average of 69 citations per article, with a range of citations from a minimum of 25 to a high of 204. The data of periodic growth of articles and citations specifies an intricate interchange between the volume of top-cited research and its impact on our study. The substantial growth in publications coupled with a decline in average citation impact suggests that, while research activity is increasing, the ability of individual articles to command attention and citations may be diminishing. Further exploration into these trends could provide valuable insights into the dynamics of academic publishing and research impact. Aksoy et al. examined the 877 articles on micro-CT endodontics published from 1995 to 2000, and more than half of the articles were published during the last six years [22].

In our study, nonclinical studies counted 72 and gained slightly higher citation impact than clinical studies. According to the analysis of LoE in our study, although there are many articles with lower levels of evidence, particularly LoE-IV, the citation impact varies greatly. LoE-II had the greatest impact, which may be a sign of the caliber or applicability of research at this level. A study on guided endodontics stated that case reports (LoE-IV) had been the most common study design [23], while another study that analyzed the top-cited articles on regenerative endodontics quantified that in vitro studies were the most common study design, followed by reviews [24]. The study by Mishra et al. on the 10 most cited articles on the management of fracture instruments stated that the majority of articles followed the clinical research studies [25]. Another study on the top-cited articles on endodontics exposed that about one-third (n = 30) of articles belonged to clinical research studies, and most of the articles followed the basic research studies. The common study designs were Review and Case series [14]. Another study on “traumatic dental injuries” revealed that 75% of the articles were related to clinical research studies [18].

The study on the 100 top-cited articles on periodontics in the Arab world stated that these articles published in the 27-year span from 1995 to 2021 received an average of 92.18 citations each, with citations ranging from a minimum of 54 to a maximum of 396 [11]. Adnan and Ullah analyzed the 100 top-cited articles on regenerative endodontics published from 1991 to 2018 [24]. The majority of articles were published in 2014, and 51% of articles originated from the United States. Another study focused on trends and characteristics of 85 articles related to guided endodontics. These articles were cited with an average of 14.84 citations per article [23].

The information about the publication sources in our study shows a distinct hierarchy in journal performance according to metrics for article output and citations. Although publications with fewer articles can nevertheless have a significant impact, the Journal of Endodontics stands out as a leader. This analysis demonstrates the variety of dental research publications and citation procedures. The top-cited articles on regenerative endodontics and guided endodontics are endorsed by the fact that most of the articles were published in the Journal of Endodontics [23,24]. Fardi et al. investigated the top-cited articles published in endodontics journals; most of the articles were published in the Journal of Endodontics, followed by Oral Surgery Oral Medicine Oral Pathology Oral Radiology and Endodontology [14]. The bibliometric study on micro-CT endodontics indicated that almost 50% of articles were published in the Journal of Endodontics and International Endodontic Journal [22].

The findings of our study about research productivity and citation effect vary by country. Even though the United States produces the most publications overall, countries like Israel and The Netherlands have higher citation impacts, demonstrating that their research has been well regarded. A deeper understanding of these processes may be possible with more investigation into certain papers and study topics. Alrubaig et al. reported that 17,879 endodontics-related papers were published worldwide between 2010 and 2022. Brazil produced the most papers (15.90%), followed by the United States (12.33%), China (5.31%), India (5.31%), and Turkey (5.08%). The research produced by England had a significant impact on citations [26]. Another bibliometric study focused on guided endodontics reported that Brazil was found to be the most productive country, closely followed by the United States [23]. Adnan and Ullah investigated the top-cited articles on regenerative endodontics and reported that 51% of articles originated from the United States [24]. Mishra et al. evaluated the bibliometric indicators of the 10 most cited articles on the management of fracture instruments. These articles were contributed by Australia, Israel, Switzerland, the United States, and Germany [25]. Fardi et al. stated in the top-cited articles on endodontics that most of the research (n = 52) came from the United States, followed by Sweden (n = 13) and England (n = 7) [14]. Another bibliometric study focused on micro-CT endodontics testified that Brazil and the University of Sao Paulo emerged as the most productive country and institution, respectively. The research produced by Switzerland and Israel had the highest citation impact, examining the research on “traumatic dental injuries,” and reported that Brazil produced most of the research, followed by the United States. Israel ranked eighth with 2.93% of the total research [18,22].

Our study highlighted that Tel Aviv University had the most articles overall; institutions such as the Academic Center for Dentistry Amsterdam demonstrate that even smaller outputs can have a significant impact on research. Additional investigation into certain study topics and citation patterns may provide insightful information about academic output and impact. A study of the 10 most cited articles on the management of fracture instruments stated the University of Melbourne was found to be the most productive institution [25]. Another study on the top-cited articles published in endodontics journals revealed that the authors affiliated with Loma Linda University produced most of the articles [14].

Significant contributions to their field are highlighted in the analysis of top authors, with a particular emphasis on Tel Aviv University. A small number of authors’ high citation impacts suggest that the quantity of publications is not always a good indicator of the quality of the study. Gaining knowledge of these dynamics can help one identify research trends, institutional strengths, and possible areas for fieldwork or investigation. Another study on the top-cited articles on guided endodontics stated that the University of Basel of Switzerland emerged as the most productive institution [23].

In the current study, the analysis of the collaboration network (Figure 2) revealed that 138 keywords are connected in a co-occurrence network. The thickness of the linkages signifies the number of collaborations, that is, a thicker linkage corresponds to a greater number. The size of the node indicates the level of cooperation; larger nodes signify greater collaboration [27]. Keywords with more occurrences have a noticeably higher total link strength, suggesting that they not only show up frequently but also have strong connections with other pertinent terms in the study. CBCT and VRF, for instance, may be related to research aimed at diagnosis and therapy. The information makes it evident that VRFs and related diagnostic methods, including CBCT, are the main focus. The diverse range of keyword occurrences in endodontics shows well-established subjects and new fields of study. These keywords are not just prevalent but also related, as indicated by the strong link strengths, which point to an integrated approach to this field's research. Fardi et al. revealed in their study on top-cited articles on endodontics that “endodontic microbiology” (n = 17) was found to be the most preferred field of study [14]. The bibliometric study on tooth intentional replacement identified future research themes, and “managing vertical root fractures” is one of them [17]. Another bibliometric study on “traumatic dental injuries” exposed that avulsion was found to be the most common dental injury (21%), followed by crown fracture (9.71%) and root fracture (5.90%) [18].

Although there was a general upward trend in top-cited articles in the last decade, the output and share of developing countries were low. Low-income countries require cooperation, education, and intellectual support. The proportion of dental scholarship programs for low-income countries can be increased by the United States, England, and other developed nations. These connections would strengthen the prosperity, health, and sustainable development of the world.

Limitations

The current study's dataset is restricted to documents that are indexed by WOS. The dataset obtained from WOS, PubMed, and Scopus can be integrated into future research to examine the larger body of knowledge regarding VRFs. The productivity of the writers' work as principal or corresponding authors was not measured in this study. These bibliometric features can be thoroughly examined in future research. The ratio of self-citations of authors was not observed; instead, only the citation metric of several bibliometric aspects of the VRFs study was observed; thus, more research is needed to assess self-citation behavior.

## Conclusions

The top-cited articles in VRFs have been found and examined by the authors in order to outline the dominant research trends and advancements in this quickly growing area of dentistry. There has been a notable increase in the number of highly cited publications (n = 52) about VRFs within the past 13 years (2010-2022). The United States stands out among the top countries due to its dominant overall research output. The majority of top-cited scientific literature on VRFs has been published in the Journal of Endodontics. In the fields of endodontics, oral surgery, and restorative dentistry, this information would be helpful to researchers, practitioners, and academics.

## Appendices

**Table 7:** List of 100 most cited articles CT: computed tomography; CBCT: cone-beam computed tomography; MTA: mineral trioxide aggregate

Serial no.	Description of the article	Total citations	Citation density by year (rank)
1.	An evaluation of endodontically treated vertically fractured teeth [28]	204	7.85 (14)
2.	Detection of vertical root fractures in endodontically treated teeth by a cone beam computed tomography scan [29]	193	12.06 (5)
3.	An in vitro study of the fracture resistance and the incidence of vertical root fracture of pulpless teeth restored six post-and-core systems [30]	180	6.92 (17)
4.	Diagnosis and possible causes of vertical root fractures [31]	180	4.00 (45)
5.	The relationship of root canal enlargement to finger-spreader induced vertical root fracture [32]	170	6.07 (23)
6.	Patterns of vertical root fracture: factors affecting stress distribution in the root canal [33]	157	7.14 (16)
7.	Prevalence of vertical root fractures in extracted endodontically treated teeth [34]	140	5.38 (27)
8.	Diagnosis of vertical root fractures in restored endodontically treated teeth: a time-dependent retrospective cohort study [35]	137	15.22 (3)
9.	Diagnosis and treatment of vertical root fractures [36]	136	3.24 (56)
10.	Comparison of five cone beam computed tomography systems for the detection of vertical root fractures [37]	134	8.93 (8)
11.	Diagnosis of vertical root fractures in endodontically treated teeth based on clinical and radiographic indices: a systematic review [38]	131	8.73 (9)
12.	Vertical root fractures in endodontically treated teeth: a clinical survey of 36 cases [39]	129	4.03 (44)
13.	Potential relationship between design of nickel-titanium rotary instruments and vertical root fracture [40]	126	8.40 (12)
14.	An evaluation of endodontically treated vertical root fractured teeth: impact of operative procedures [41]	124	5.17 (31)
15.	Evaluation of an artificial intelligence system for detecting vertical root fracture on panoramic radiography [42]	119	23.80 (2)
16.	Iatrogenic vertical root fractures in endodontically treated teeth [43]	107	2.89 (61)
17.	Vertical root fracture in endodontically versus nonendodontically treated teeth: a survey of 315 cases in Chinese patients [44]	106	4.08 (43)
18.	A demographic analysis of vertical root fractures [45]	105	5.53 (26)
19.	Diagnosis of vertical root fractures by cone-beam computed tomography in root-filled teeth with confirmation by direct visualization: a systematic review and meta-analysis [46]	101	25.25 (1)
20.	The histopathogenesis of vertical root fractures [47]	101	2.46 (70)
21.	Detection of vertical root fractures by using cone beam computed tomography with variable voxel sizes in an in vitro model [48]	96	6.86 (18)
22.	Vertical root fractures: clinical and radiographic diagnosis [49]	96	4.36 (39)
23.	A comparison of cone beam computed tomography and periapical radiography for the detection of vertical root fractures in nonendodontically treated teeth [50]	95	8.64 (10)
24.	Detection of vertical root fractures by using cone-beam computed tomography: a clinical study [51]	95	6.79 (19)
25.	Load and strain during lateral condensation and vertical root fracture [52]	94	3.62 (49)
26.	Evaluation of cone-beam computed tomography in the diagnosis of vertical root fractures: the influence of imaging modes and root canal materials [53]	93	8.45 (11)
27.	Detection of vertical root fractures by conventional radiographic examination and cone beam computed tomography - an in vitro analysis [54]	91	7.58 (15)
28.	Vertical root fractures [55]	88	2.51 (68)
29.	Further investigation of spreader loads required to cause vertical root fracture during lateral condensation [56]	87	2.29 (77)
30.	Dental vertical root fractures: value of CT in detection [57]	84	3.23 (57)
31.	Detection of vertical root fractures in intact and endodontically treated premolar teeth by designing a probabilistic neural network: an ex vivo study [58]	81	10.13 (6)
32.	Cone-beam computed tomography for detecting vertical root fractures in endodontically treated teeth: a systematic review [59]	81	9.00 (7)
33.	Evaluation of three imaging techniques for the detection of vertical root fractures in the absence and presence of gutta-percha root fillings [60]	80	6.15 (22)
34.	The detection of vertical root fractures in root filled teeth with periapical radiographs and CBCT scans [61]	79	6.58 (20)
35.	Diagnosis of vertical root fractures with optical coherence tomography [62]	79	4.65 (35)
36.	Detection of vertical root fractures of different thicknesses in endodontically enlarged teeth by cone beam computed tomography versus digital radiography [63]	78	5.20 (29)
37.	Finite element analysis and strain-gauge studies of vertical root fracture [64]	76	3.45 (51)
38.	Role of cone-beam computed tomography in diagnosis of vertical root fractures: a systematic review and meta-analysis [1]	73	8.11 (13)
39.	Detection of vertical root fractures in vivo in endodontically treated teeth by cone-beam computed tomography scans [65]	72	5.54 (25)
40.	Diagnosis and management of teeth with vertical root fractures [66]	71	2.73 (64)
41.	Detection of vertical root fracture using cone-beam computerized tomography: an in vitro assessment [67]	69	4.60 (36)
42.	Vertical root fractures and dentin defects: effects of root canal preparation, filling, and mechanical cycling [68]	68	5.23 (28)
43.	Vertical root fracture in nonendodontically treated teeth [69]	66	2.20 (82)
44.	An in vitro study of spreader loads required to cause vertical root fracture during lateral condensation [70]	65	1.55 (92)
45.	Influence of the artefact reduction algorithm of Picasso Trio CBCT system on the diagnosis of vertical root fractures in teeth with metal posts [71]	64	6.40 (21)
46.	Vertical root fracture and root distortion: effect of spreader design [72]	63	1.75 (90)
47.	Effect of new obturating materials on vertical root fracture resistance of endodontically treated teeth [73]	59	3.28 (55)
48.	Radiographic features of vertically fractured, endodontically treated maxillary premolars [74]	58	2.23 (78)
49.	Comparing the in vivo diagnostic accuracy of digital periapical radiography with cone-beam computed tomography for the detection of vertical root fracture [75]	57	5.18 (30)
50.	Comparison of digital with conventional radiography in detection of vertical root fractures in endodontically treated maxillary premolars: an ex vivo study [76]	57	3.35 (52)
51.	Detection of artificially induced vertical radicular fractures using tuned aperture computed tomography [77]	57	2.38 (73)
52.	Prevalence of vertical root fracture as the reason for tooth extraction in dental clinics [78]	56	5.60 (24)
53.	Radiographic features of vertically fractured endodontically treated mesial roots of mandibular molars [79]	55	2.89 (60)
54.	Vertical root fracture in nonendodontically treated teeth--a clinical report of 64 cases in Chinese patients [80]	55	2.04 (84)
55.	Detection of vertical root fractures in the presence of intracanal metallic post: a comparison between periapical radiography and cone-beam computed tomography [81]	53	4.42 (38)
56.	Effects of root canal sealers on vertical root fracture resistance of endodontically treated teeth [82]	51	2.22 (80)
57.	Pattern of bone resorption in vertically fractured, endodontically treated teeth [83]	48	1.92 (86)
58.	Vertical fracture of root filled teeth restored with posts: the effects of patient age and dentine thickness [84]	46	3.07 (59)
59.	Three-dimensional, non-destructive visualization of vertical root fractures using flat panel volume detector computer tomography: an ex vivo in vitro case report [85]	46	2.30 (76)
60.	Comparison of mandibular premolars and canines with respect to their resistance to vertical root fracture [86]	46	2.19 (83)
61.	Vertical root fracture treated by bonding fragments and rotational replantation [87]	44	1.91 (87)
62.	Periodontal healing after bonding treatment of vertical root fracture [88]	44	1.83 (88)
63.	Present status and future directions: vertical root fractures in root filled teeth [9]	41	13.67 (4)
64.	Influence of exposure parameters on the detection of simulated root fractures in the presence of various intracanal materials [89]	41	5.13 (32)
65.	Cone beam computed tomography for the diagnosis of vertical root fractures: a systematic review of the literature and meta-analysis [90]	41	3.73 (47)
66.	Spreader load required for vertical root fracture during lateral compaction ex vivo: evaluation of periodontal simulation and fracture load information [91]	41	2.41 (72)
67.	Clinical and radiographic characteristics of vertical root fractures in endodontically and nonendodontically treated teeth [92]	40	5.00 (33)
68.	Vertical root fracture in endodontically treated teeth: a review of 25 cases [93]	40	1.67 (91)
69.	Vertical root fracture in upper premolars with endodontic posts: finite element analysis [94]	38	2.38 (74)
70.	Performance of an artefact reduction algorithm in the diagnosis of in vitro vertical root fracture in four different root filling conditions on CBCT images [95]	37	4.11 (42)
71.	Vertical root fracture and relative deformation during obturation and post cementation [96]	37	1.09 (95)
72.	Alveolar bone loss associated with vertical root fractures. Report of six cases [97]	37	0.79 (97)
73.	Performance of an artificial neural network for vertical root fracture detection: an ex vivo study [98]	34	2.83 (62)
74.	The effect of isthmus on vertical root fracture in endodontically treated teeth [99]	33	3.30 (53)
75.	Influence of CBCT enhancement filters on diagnosis of vertical root fractures: a simulation study in endodontically treated teeth with and without intracanal posts [100]	33	3.30 (54)
76.	Periodontal destruction associated with vertical root fracture: report of four cases [101]	33	0.69 (99)
77.	Vertical root fracture: factors related to identification [102]	32	4.00 (46)
78.	Assessment of vertical root fractures using three imaging modalities: cone beam CT, intraoral digital radiography and film [103]	32	2.46 (71)
79.	Repair of incomplete vertical root fractures in endodontically treated teeth--in vivo trials [104]	32	1.10 (94)
80.	Diagnosis of mesiodistal vertical root fractures in teeth with metal posts: influence of applying filters in cone-beam computed tomography images at different resolutions [105]	31	4.43 (37)
81.	The influence of metallic posts in the detection of vertical root fractures using different imaging examinations [106]	31	2.82 (63)
82.	Vertical root fracture: prevalence, etiology, and diagnosis [107]	31	2.58 (66)
83.	Diagnosis and treatment of endodontically treated teeth with vertical root fracture: three case reports with two-year follow-up [8]	31	2.21 (81)
84.	Comparative diagnostic yield of cone beam CT reconstruction using various software programs on the detection of vertical root fractures [108]	30	2.50 (69)
85.	Accuracy of detecting vertical root fractures in non-root filled teeth using cone beam computed tomography: effect of voxel size and fracture width [109]	29	4.83 (34)
86.	Detection of artificially induced vertical root fractures of different widths by cone beam computed tomography in vitro and in vivo [110]	29	3.22 (58)
87.	Surgical management of vertical root fractures for posterior teeth: report of four cases [111]	29	2.23 (79)
88.	Vertical root fractures in adjacent maxillary premolars: an endodontic-prosthetic perplexity [112]	29	1.07 (96)
89.	Optimization of tube current in cone-beam computed tomography for the detection of vertical root fractures with different intracanal materials [113]	28	3.50 (50)
90.	Analysis of the width of vertical root fracture in endodontically treated teeth by 2 micro-computed tomography systems [114]	28	2.55 (67)
91.	Effects of root canal preparation, various filling techniques and retreatment after filling on vertical root fracture and crack formation [115]	27	2.70 (65)
92.	Comparative evaluation of rotary ProTaper, Profile, and conventional stepback technique on reduction in Enterococcus faecalis colony-forming units and vertical root fracture resistance of root canals [116]	27	1.80 (89)
93.	In vivo detection of subtle vertical root fracture in endodontically treated teeth by cone-beam computed tomography [117]	26	4.33 (40)
94.	Diagnosis of vertical root fracture in teeth close and distant to implant: an in vitro study to assess the influence of artifacts produced in cone beam computed tomography [118]	26	4.33 (41)
95.	Influence of voxel size on cone-beam computed tomography-based detection of vertical root fractures in the presence of intracanal metallic posts [119]	26	3.71 (48)
96.	Resistance to vertical fracture of MTA-filled roots [120]	26	2.36 (75)
97.	A successful treatment of vertical root fracture: a case report and 4-year follow-up [121]	26	1.53 (93)
98.	Different representations of vertical root fractures detected by cone-beam volumetric tomography: a case series report [122]	25	1.92 (85)
99.	A method for producing experimental simple vertical root fractures in dog teeth [123]	25	0.78 (98)
100.	Treating vertical root fractures [124]	25	0.61 (100)
